# Leaky Gut in IBD: Intestinal Barrier–Gut Microbiota Interaction

**DOI:** 10.4014/jmb.2203.03022

**Published:** 2022-06-30

**Authors:** Shunying Yu, Yibin Sun, Xinyu Shao, Yuqing Zhou, Yang Yu, Xiaoyi Kuai, Chunli Zhou

**Affiliations:** Department of Gastroenterology, The Affiliated Suzhou Hospital of Nanjing Medical University, Suzhou Municipal Hospital, Gusu School, Nanjing Medical University, Suzhou 215001, Jiangsu, P.R. China

**Keywords:** Inflammatory bowel disease, intestinal barrier, gut microbiota, neuroimmune system

## Abstract

Inflammatory bowel disease (IBD) is a global disease that is in increasing incidence. The gut, which contains the largest amount of lymphoid tissue in the human body, as well as a wide range of nervous system components, is integral in ensuring intestinal homeostasis and function. By interacting with gut microbiota, immune cells, and the enteric nervous system, the intestinal barrier, which is a solid barrier, protects the intestinal tract from the external environment, thereby maintaining homeostasis throughout the body. Destruction of the intestinal barrier is referred to as developing a “leaky gut,” which causes a series of changes relating to the occurrence of IBD. Changes in the interactions between the intestinal barrier and gut microbiota are particularly crucial in the development of IBD. Exploring the leaky gut and its interaction with the gut microbiota, immune cells, and the neuroimmune system may help further explain the pathogenesis of IBD and provide potential therapeutic methods for future use.

## Introduction

Inflammatory bowel disease (IBD), which includes ulcerative colitis (UC) and Crohn’s disease (CD), is a global disease with the highest incidence in Western countries, although the incidence is increasing in newly industrialized countries [1]. The annual incidence of UC ranges from 0–19.2 per 100,000 individuals in North America and 0.6–24.3 per 100,000 in Europe. The prevalence of CD is 0–20.2 per 100,000 individuals in North America and 0.3–12.7 per 100,000 in Europe [2]. The incidence of IBD in Asia is 1.4 per 100,000 people. India has the highest incidence of IBD, at 9.3 per 100,000. Nowadays, IBD has risen from the fifth leading cause of death due to gastrointestinal disease in 1990 to the fourth leading cause in 2017. Owing to the long course of the disease and the difficulties associated with treatment, IBD seriously affects the quality of the patient’s life. Thus, exploring the pathogenesis and effective treatment for IBD in depth is urgent [3].

The gut is the largest interface between the body and the external environment. Maintaining a stable intestinal barrier is essential to preventing luminal substances and pathogens from entering the internal environment. Thousands of microbes in the intestine are not only challenges to IBD, but also to future treatment options. The gut microbiota can protect against the development of IBD, suggesting that regulation of the gut microbiota could also be a future treatment option. IBD patients often exhibit dysbiosis, as confirmed by the results from fecal and mucosal microbial analyses. Intestinal dysbiosis and IBD are often mutually causal and persistent dysbiosis leads to long-standing and severe inflammation. However, some commensal bacteria improve IBD and may be a future treatment option.

The intestinal mucosal barrier, which consists of intestinal epithelial cells and intercellular tight junctions, plays a significant role in maintaining intestinal homeostasis and resisting intestinal infection. By interacting with the gut microbiota, immune cells, and exosomes, the mucosal barrier forms a solid barrier between the intestinal tract and the external environment, thereby maintaining intestinal homeostasis. The enteric nervous system (ENS) is sometimes called the “second brain,” as it can regulate intestinal motility and secretion independent of the brain itself. The interactions between the ENS, gut immunity, and the gut microbiota are indispensable to maintaining intestinal barrier function and preventing disease progression. A breached intestinal barrier allows microbes unlimited access to the lamina propria and the bloodstream [4], a condition referred to as “leaky gut.” IBD patients often suffer from abnormal intestinal permeability and enteric blood barrier impairment [5]. Destruction of the intestinal barrier is one characteristic of IBD [6, 7]. Thus, studying the interactions between the gut microbiota, gut immunity, ENS, and the intestinal barrier may help further explore the pathogenesis of IBD and provide options for future treatment.

## Gut Microbiota Changes in IBD: Challenges and Options

The gut microbiota mainly consists of Bacteroidetes, Firmicutes, Actinobacteria, Proteobacteria, and Verrucomicrobia [8]. Dysbiosis, which refers to changes in the gut microbiota population, diversity, location, and abundance, is a main characteristic of IBD [9]. Changes in the gut microbiota are related to IBD location, duration of disease, and genetic risk score [10]. Fecal microbial richness is an average of 25% lower in IBD patients than in healthy people [11]. In fecal samples from patients with IBD, Bacteroidetes, Proteobacteria, *Enterobacteriaceae* family [12], *Fusobacterium*, - and *Enterococcus faecalis* [13] are significantly increased, while Bifidobacteria and butyrate-producing bacteria such as *Faecalibacterium*, *Eubacterium*, *Roseburia*, *Lachnospiraceae* and *Ruminococcaceae* are decreased compared with healthy individuals [14, 15]. Actinobacteria and Tenericutes are decreased in CD patients, and Firmicutes is decreased in UC patients compared with healthy individuals. *Roseburia intestinalis* (*R. intestinalis*), which is abundant in the intestine and enables the production butyrate, is decreased in IBD patients compared with healthy individuals, and is related to a higher number of IBD risk alleles [10] (Table 1).

In a healthy state, the mucus layer separates epithelial cells from bacteria. Most local symbiotic bacteria living in the intestinal lumen do not make direct contact with epithelial cells [16, 17]. However, IBD patients present with a high density of mucosa-associated bacteria originating from biofilms containing a large number of *Bacteroides fragilis* (*B. fragilis*) and a substantial amount of extracellular matrix, indicating that the microbiota is more toxic and biofilms are more invasive in patients with IBD than in healthy individuals [18, 19].

Dysbiosis and IBD are often mutually causal. Sustained and severe dysbiosis seriously exacerbates IBD. Some commensal bacteria can help improve IBD and are expected to become new treatment options in the future.

In mice with dextran sulfate sodium (DSS)-induced colitis, *Feacalibacterium prausnitzii* (*F. prausnitzii*) enhanced intestinal barrier function and reduced disease severity by ameliorating paracellular permeability [20]. In another study, Caco-2 and HT29-MTX cells, stimulated with IL-1β, TNF-α, IFN-γ, and LPS, were used to mimic the inflammatory environment seen in IBD patients. *F. prausnitzii*, *R. intestinalis*, and *Bacteroides faecis* improved the intestinal barrier function and epithelial barrier integrity by increasing transepithelial electrical resistance (TEER), a marker of tight junction integrity, and thereby alleviating paracellular permeability of the cell monolayer and reducing paracellular leakage induced by inflammation in separation or in combination [21]. Similarly, *Lactobacillus rhamnosus* CNCM I-3690 partially restored intestinal barrier function and increased expression of the tight junction proteins occludin and E-cadherin in a monolayer of Caco-2 cells stimulated with TNF-α and in a mouse model of increased colonic permeability [22].

*Akkermansia muciniphila* (*A. muciniphila*) is a gram-negative and strictly anaerobic bacterium belonging to Verrucomicrobia [23], whose abundance is decreased in animal models of colitis and in patients. *A. muciniphila* significantly improves histological injuries in the colon and over-activated immune responses in mice with acute colitis by reducing the numbers of infiltrating macrophages and CD8^+^ cytotoxic T lymphocytes in the colon [24, 25]. β-N-acetylhexosaminidase Amuc_2109 produced by *A. muciniphila* prevents DSS-induced colitis in mice by strengthening the intestinal barrier and regulating the gut microbiota. The specific mechanisms include improving epithelial cell structure, supplementing goblet cells, and reducing inflammatory cell infiltration [26]. *A. muciniphila* significantly restores LPS levels, inhibits intestinal inflammation, and increases the expression of genes encoding barrier-forming tight junction proteins (CLDN3 and 4) in mice fed a high-fat diet [27]. *A. muciniphila* also improves age-associated colon mucus thinness and changes in inflammation and immune mediators. In addition, the abundance of *A. muciniphila* is inversely correlated with obesity and type 2 diabetes, suggesting that it plays a significant role in regulating chronic metabolic and inflammatory disorders [28, 29].

Intestinal inflammation promotes the proliferation of fungi, which can in turn regulate intestinal inflammation. *Candida albicans* (*C. albicans*) plays a pro-inflammatory role and aggravates the development of IBD. *Lactobacillus rhamnosus* L34 antagonizes *C. albicans* and alleviates IBD severity by reducing intestinal barrier damage, ameliorating intestinal and systemic inflammation, and improving dysbiosis [30]. Probiotics such as *Lactobacillus reuteri* ATCC 55730, Bifidobacterium triple viable agents, or a multi-strain probiotic combined with conventional IBD drugs such as glucocorticoid and mesalazine show a greater ability than monotherapy to alleviate inflammatory responses, upregulate the expression of intestinal barrier-related proteins, and promote mucosal homeostasis [31-34]. *Bifidobacterium adolescentis* was shown to reinforce the intestinal barrier by enhancing mucus thickness and increasing occludin, Muc2, and Muc3 mRNA expression in a mouse model of chronic colitis [35].

IBD is often accompanied by dysbiosis, and persistent dysbiosis reciprocally aggravates inflammation. Some commensal intestinal bacteria improve IBD by regulating the intestinal barrier and immune responses, which could represent a novel, future treatment option. Therefore, exploring changes in the gut microbiota that occur in patients with IBD could further elucidate the pathogenesis of IBD and provide better treatments for IBD.

## The Barrier Function of the Intestinal Epithelium in IBD 

The intestinal epithelium is lined by a single layer of intestinal epithelial cells (IECs), which form a tight and selective barrier. The integrity of epithelial cells is mediated by tight junction proteins such as occludin, claudin, junctional adhesion molecule, and tricellulin [21].

O-linked glycoproteins, called mucins, are the main components of the mucus barrier [36]. In glycan-defective mice, colonic mucosa-associated commensal microorganisms are deficient and intestinal mucosal permeability, colonic bacteria levels, and susceptibility to IBD are increased, leading to severe spontaneous colitis [37, 38]. Strong alterations in glycan pattern are often accompanied by a more severe disease course [37, 39]. In remission, Muc2 O-glycosylation distribution gradually became normal, suggesting that glycosylation profiling could be used to predict disease progression [40].

The mucus layer is the natural and selective habitat of the gut microbiota, which in turn affects mucus composition, promotes mucus secretion, and increases mucus thickness [41, 42]. Compared with the inner layer of mucus, which is firm and contains few bacteria, the outer layer is thick and loose and contains many bacteria [43, 44]. Dietary fiber serves as an energy source for intestinal bacteria. In a fiber-free diet condition, Muc2 polysaccharides are consumed by bacteria, resulting in thinning of the inner mucous layer [45] and bacterial invasion into the lamina propria, which ultimately promotes IBD development [46]. Missense mutation of Muc2 in Winnie mice results in abnormal Muc2 synthesis, depletion of the mucus layer, and spontaneous development of moderate colitis with epithelial erosions and immune cell infiltration, which closely mimics the condition of patients with UC. Prior to intestinal histological changes, dysbiosis had already established in 4-week-old mice. The genera *Alkaliphilus*, *Candidatus*, *Blochmannia*, and *Caldilinea* exhibited the largest populations, and *Deferribacteres* abundance was significantly lower than in WT mice during the observation period [47]. Commensal and pathogenic bacteria are involved in the regulation of mucin production [48]. A study comparing the intestinal microbiota and mucus phenotype of mice that were raised in the same SPF environment but in different cages found that the number of *Erysipelotrichi* increased in mice whose mucus layer was difficult to penetrate. Meanwhile, the levels of Proteobacteria and TM7 bacteria increased in the mucus of the distal colon, which in comparison has an easy-to-penetrate mucus layer [49]. Although mucus organization in the colon of GF mice is similar to that seen in conventionally raised mice, the internal mucus layers in GF mice are more susceptible to bacteria-sized beads [50].

Dysregulation of intracellular tight junctions leads to epithelial translocation of microorganisms, uncontrolled antigen presentation, and an overactive immune response that is related to chronic inflammation [7]. Abnormal intestinal permeability precedes the onset of CD by 3 years and may be an important component of pathogenesis. Increased intestinal permeability in asymptomatic individuals is significantly associated with the risk of developing CD [51].

The permeability of the ileum and colon increases prior to the onset of intestinal inflammation in IL10^-/-^ mice. However, in a pathogen-free environment, neither abnormal inflammation nor increased intestinal permeability occurs in these mice. TNF-α and IL-1β stimulate gel-forming mucins [52], and Th2 cytokines participate in mucin gene expression, upregulating Muc2 and Muc5AC by binding to the IL-4 receptor. By inhibiting endoplasmic reticulum stress and promoting intestinal mucus production, IL-10 prevents immature mucin production and inflammation [53, 54].

Exosomes act as intercellular messengers and significantly affect IEC-gut microbiota-immune system interactions. Administration of exosomes directly regulates IEC proliferation, differentiation, and tightly connected molecules of IECs, reducing disease severity and preventing IBD recurrence [55, 56]. Exosome-like nanoparticles (ELNs) derived from ginger (including the microRNA mdomiR7267-3p) are preferentially absorbed by *Lactobacillus rhamnosus* (*L. rhamnosus*, LGG). MicroRNA mdomiR7267-3p downregulates the LGG monooxygenase ycnE, promotes indole-3-carboxaldehyde (I3A) production, and induces IL-22 via the AHR-mediated pathway, thus strengthening the intestinal barrier and alleviating colitis in mice [57]. Mesenchymal stem cell (MSC)-derived exosomes alleviate epithelial cell inflammation induced by LPS in vitro and upregulate the expression of tight junction proteins. In an animal model of colitis, they improved barrier function by reducing the levels of TNF-α, IL-6, IL-1β, IL-17, and other cytokines, upregulating the levels of intestinal barrier markers claudin-1 and ZO-1, increasing *Lactobacillus* abundance, and inhibiting Bacteroides abundance, thereby restoring gut microbiota composition and alleviating experimental colitis in mice [58]. MiR-146b originating from DC-derived exosomes (DC-Exs) regulates the NF-κB pathway to improve intestinal barrier function [59, 60]. Exosomal proteins such as glucose-regulated protein 78 (GRP78), annexin-1 (ANXA-1) and cellular prion protein (PRPC) also participate in repairing and maintaining intestinal mucosal barrier integrity and function [61, 62].

Maintenance of the mucus barrier is closely related to the gut microbiota, immunity, and genetic changes. Intestinal barrier defects may occur prior to intestinal inflammation and result in a dysregulated epithelial barrier response toward normal gut microbiota in these mice [63]. Thus, studying the relationship between the intestinal epithelium and the gut microbiota in IBD may help provide a better understanding of the role of the intestinal barrier in IBD and possibly uncover ways to use it as a target for the treatment of IBD.

## The Role of Intestinal Neuroimmunity in IBD 

The gut contains the largest amount of lymphoid tissue in the human body and a wide range of nervous system components to ensure intestinal homeostasis and function. Apart from the brain, the ENS is the most complex nervous system in the body as it interacts with intestinal immunity and the gut microbiota. Increasing evidence demonstrates that the intestinal nervous system–immune system interaction is essential to maintaining intestinal barrier function. Thus, exploring the role of intestinal neuroimmunity contributes to further clarification of the pathogenesis of IBD.

Macrophages are the most abundant leukocytes in the lamina of healthy intestine [64]. They are closely related to IECs and are regulated by the gut microbiota [65, 66]. Fungi are abundant in the distal colon and can induce intestinal epithelial cell apoptosis. A mouse study involving fluid absorption of fungi metabolites confirmed that macrophages helped terminal colonic epithelial cells quickly recognize and prevent the absorption of toxic fluids, thus maintaining epithelial integrity and local homeostasis [67]. In mice with macrophage deficiency, a large number of colonic epithelial cells are apoptotic, and intestinal permeability is increased [68-70]. The integrity of the subepithelial band of indigenous macrophages in the lamina propria is crucial for the host’s health. The SLP band of CD68^+^ macrophages is the first line of defense against gut microbiota intrusion into the lamina propria mucosae. In UC and CD, disruption of the subepithelial localization of macrophages leads to the loss of intestinal barrier integrity [71]. The SLP band of CD68^+^ macrophages is randomly distributed in the noninflammatory tissue of patients with IBD and microscopic colitis, but exhibits continuous distribution in the colon of patients without IBD. In patients with UC in remission (UCre) and right-sided Crohn’s colitis (RCC), the SLP band of CD68^+^ macrophages was fragmented to totally abrogated [72].

Innate lymphoid cells (ILCs), whose functions are relevant to the innate immune system, are distributed throughout the gastrointestinal tract [73]. ILCs secrete IL-22, regulate the anatomic location of lymphoid symbiotic bacteria, inhibit the growth of pathogenic bacteria, and prevent bacteria from damaging the intestinal tissue [74, 75]. Murine enteric ILC3s express the neuroregulator tyrosine kinase receptor RET. ILC3s can be activated by IL-1β secreted by macrophages and in turn activate macrophages by secreting GM-CSF. Mice with RET-deficient ILC3s exhibit impaired IL-22 production, reduced epithelial reactivity and increased susceptibility to *C. rodentium* infection [76]. Dysregulation of brain rhythmicity led to disrupted circadian oscillations in ILC3 expression, impaired epithelial reactivity, dysbiosis and increased susceptibility to intestinal infection [77]. EGC-derived glial-derived neurotrophic factor family ligands (GFLs) activate RET-induced IL-22 secretion from ILC3s by neuroregulatory signaling, thereby mediating intestinal repair. In addition, neurotrophic factor directly regulates IL-22 downstream of STAT3 activation, which is the key factor in maintaining epithelial integrity and promoting intestinal regeneration [76].

The gut microbiota stimulates macrophages to secrete IL-1β and then induce RORγt^+^ ILCs to produce IL-22 [78]. RORγt^+^ ILCs can be stimulated by IL-25 derived from epithelial tuft cells in a microbiota-dependent manner [79]. AhR stimulates innate intestinal immunity by regulating RORγt^+^ ILCs [80, 81]. In AhR-deficient mice, RORγt^+^ ILCs increase cell apoptosis and reduce IL-22 production, making mice susceptible to *C. rodentium* infection [82].

Mitochondria are the most sensitive organelles to microbial signals. Recent studies have confirmed that mitochondrial dysfunction is related to dysbiosis and intestinal inflammation. Toxins produced by pathogenic bacteria directly damage IEC mitochondria by suppressing mitochondrial ATP-sensitive potassium channels, causing hyperpolarization and apoptosis of the mitochondrial membrane, and damaging the intestinal barrier [83]. When *Escherichia coli* is used to mimic bacterial invasion in IBD, the mitochondria mediate occludin transfer from the perijunctional region to the cytosol, destroying tight junctions [84], which is not conducive to IEC function [85]. Moreover, colonic epithelial cells with mitochondrial dysfunction are more susceptible to barrier function damage [86].

In the early stage of CD, variations in the ultrastructure of epithelial mitochondria such as dissolved/irregular cristae precede changes in tight junctions [87]. Epithelial cells lose their tolerance to commensal bacteria when they exhibit mitochondrial dysfunction [86]. Mitochondrial depolarization increases mtROS level, which in turn destroys tight and adhesive junctions [88]. mtROS stimulates the redistribution of occludin and ZO-1 from intercellular junctions into intracellular compartments during DSS administration, leading to leakage of tight junctions and damage to barrier integrity [89].

Intestinal mucosal barrier immunity affects the intestinal barrier and inflammatory responses. The interaction between the gut nervous system and immunity also plays an important role in maintaining tissue homeostasis.

Histamine and tryptase derived from mast cells activate myenteric and submucosal neurons, respectively [90]. Peptidergic neurons regulate mast cells through production of substance P, calcitonin gene-related peptide (CGRP), and vasoactive intestinal polypeptide (VIP) [91, 92]. Intestinal muscularis macrophages (MMs), which are located in and surrounding the myenteric plexus, regulate the activity of enteric neurons and peristalsis by secreting bone morphogenetic protein 2 (BMP2) in a microbiota-dependent manner [93]. During infection with *Salmonella* Typhimurium (S.t.), extrinsic sympathetic neurons in intestinal muscularis are activated and release noradrenaline, which acts on muscularis macrophages that express β2 adrenergic receptor (β2AR). Activation of β2AR-expressing macrophages by noradrenaline drives the acquisition of a tissue-protective macrophage phenotype, especially by the induction of *Arg1* and *Chi3l3* expression, which may help preserve an anti-inflammatory milieu in the muscularis [94]. Intestinal bacterial infection can lead to persistent intestinal inflammation. A reduction in myenteric neurons due to cell death can be mediated by the inflammasome components encoded by *Nlrp6* and *Casp11*. MMs respond to enteric pathogens quickly and reduce neuronal death caused by infection via the MM-β2-adrenergic-arginase 1-polyamine axis [95].

In addition to being expressed by immune and epithelial cells, enteric neurons also express IL-18. IL-18 protects the gut from enteric bacterial pathogens such as S.t. and maintains the mucus barrier by upregulating AMP expression in the goblet cells of the intestinal epithelium. Knocking out IL-18 from immune and epithelial cells has no effect on AMP production. However, in the absence of neural-derived IL-18, AMP levels are decreased in the intestinal mucus, and mice become more susceptible to *Salmonella* infection [96, 97].

Gut-innervating nociceptor sensory neurons sense noxious stimuli and protect mammals from danger by initiating protective responses. In intestinal infection, nociceptor neurons limited the invasion points of *Salmonella* enterica serovar Typhimurium (STm) by reducing the density of microfold (M) cells in ileum Peyer’s patch (PP) follicle-associated epithelia (FAE). By maintaining levels of segmented filamentous bacteria (SFB) attached to PP and villi, nociceptors shape the small intestine microbiota and maintain ileal homeostasis [98].

The glial immune circuit consists of TLR signaling on enteric glia, activation of MYD88, GDNF and its family ligands, which are maintained by the gut microbiota. GF mice or antibiotic-treated mice lack a mucosal enteric glial network and exhibit delayed gastrointestinal motility [76, 99, 100]. Myenteric AH neurons are enteric sensory neurons. In the complete absence of bacteria, their neuronal excitability is reduced, but the effect is rescued when bacteria are restored to GF mice [101]. *B. fragilis*, LGG, and *Lactobacillus reuter* stimulate AH neurons, while *Bifidobacterium longum* (*B. longum*) reduces their excitability [102-104].

The neuroimmune system affects the occurrence and development of IBD by multiple pathways and mechanisms. The interaction between the enteric nervous and immune systems has important effects on intestinal barrier function and intestinal homeostasis. Thus, studying the neuroimmune circuits is an important future direction in the IBD field.

## Regulation of Gut Microbiota-Related Metabolites on IBD

Bile acids are synthesized in the liver and converted into secondary bile acids by microorganisms when they reach the intestinal lumen. Active conjugated bile acids are absorbed in the terminal ileum and returned to the liver via the portal vein. IBD-related dysbiosis may lead to bile acid dysmetabolism and affect intestinal homeostasis [105]. Ursodeoxycholic acid (UDCA), a component of bear bile, is cytoprotective and anti-inflammatory. Administration of UDCA inhibits excessive apoptosis and abnormal increases in epithelial permeability, maintaining barrier function and protecting against intestinal inflammation [106]. In an in vitro wound-healing experiment and a mouse model of intestinal barrier damage, UDCA induced IEC migration after acute injury and protected the intestinal barrier from damage, which prevented the early onset of necrotizing colitis, a form of enterogenous sepsis [107]. UDCA is metabolized to lithocholic acid (LCA) in the colon [108]. In an in vitro cell model induced by inflammatory factors, LCA inhibited excessive apoptosis, prevented abnormal increases in epithelial permeability, and inhibited cytokine release from the colonic epithelium [109, 110].

Short-chain fatty acids (SCFAs) are mainly produced in the cecum and the proximal colon by Bacteroidetes and Firmicutes. A decrease in intestinal SCFAs may result in increased intestinal permeability and severe inflammation [111]. Gut microbiota-derived butyrate maintains the epithelial barrier by inhibiting phosphorylation of AKT and NF-κB p65 signaling pathway in macrophages [112]. Butyrate promotes epithelial barrier function by activating transcription factors such as STAT3 and SP1 and inducing genes encoding tight junction and protein reassembly. Even when the cells are exposed to inflammatory conditions, butyrate maintains and/or increases the TEER of human Caco-2 and T_84_ cells, rat small intestine cdx2-IEC cells, and small intestine porcine IPEC-J2 cells [113-116]. Such effects can also be achieved in Caco-2 cells by treating them with supernatant containing CD microbiota supplemented with the probiotic strain *Butyricicoccus pullicaecorum* 25-3^T^ or with a mix of six butyrate producers [116, 117].

L-tryptophan (Trp) is an essential amino acid for intestinal mucosal cells that is involved in intestinal inflammation, epithelial barrier maintenance, and homeostasis [118]. Aryl hydrocarbon receptor (AhR) is abundant on the mucosal surface, enhances intestinal epithelial barrier function, and regulates the immune response when activated. Activation of the Trp-AhR pathway induces the expression of cytokines such as IL-22 and IL-17, maintains the barrier function of the intestinal mucosa, and regulates intestinal homeostasis. The expression levels of AhR, tryptophan, and its metabolites are downregulated in patients with IBD [119, 120]. FICZ6-formylindolo [3,2-b] carbazole (FICZ), which is an endogenous AhR ligand, significantly improved expression of the above mucosa-associated proteins and gradually restored TEER [121, 122]. *Lactobacilli* metabolizes Trp into the AhR ligand I3M tryptamine and promotes AhR-dependent IL-22 transcription, maintains microbial diversity, inhibits colonization by *C. albicans*, and protects the gut mucosa from inflammation [123].

**Fig. 1:**
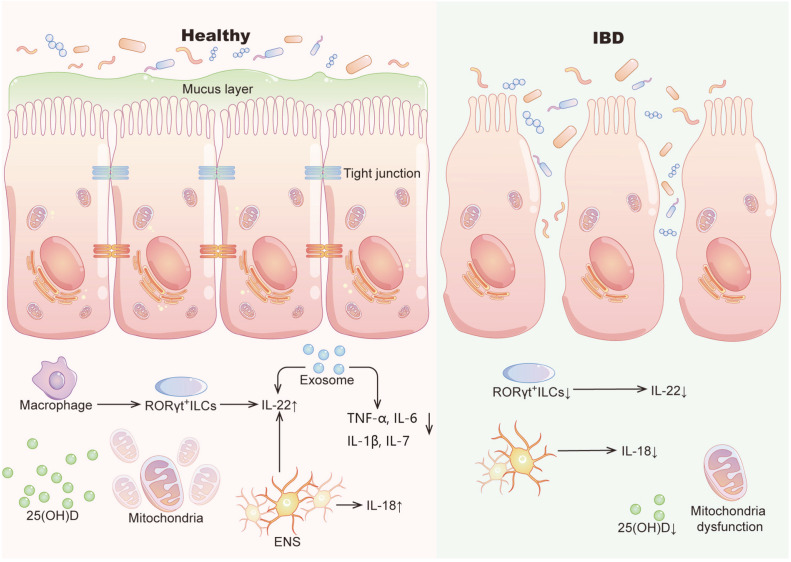
In healthy conditions, intestinal barrier is intact and is reinforced by tight junctions. Mucus forms a physical barrier between bacteria and epithelium, protecting epithelium from microbes in lumen. 25(OH) D and complete VDR signal regulate structure and function of intestinal epithelial cells, affect expression of intestinal tight junction related proteins, thus increasing intestinal barrier integrity. Affected by gut microbiota, mitochondria enhance integrity of intestinal barrier and maintain its function. Macrophages are the most abundant leukocytes in healthy intestine. Gut microbiota stimulate macrophages to secrete IL-β and then induce RORγt^+^ ILCs to produce IL-22. Enteric neurons system (ENS) is the essential source of IL-18, which drives AMP production and resists intestinal infection. IL-22 induced by EGCs mediates intestinal repair. Mesenchymal stem cell (MSC)-derived exosomes improve barrier function by reducing the levels of TNF-α, IL-6, IL-1β and IL-17 in IBD animal model. MicroRNA mdomiR7267-3p in exosome-like nanoparticles (ELNs) induces IL-22 by AHR-mediated pathway, thus improving intestinal barrier. However, in IBD, mucosal barrier and tight junction are broken down. Destruction of intestinal barrier causes bacteria to come into direct contact with epithelium and enter into systemic bloodstream unabated. 25(OH) D with protective effect is reduced. Function of mitochondria has also changed. Dissolved/ irregular cristae of mitochondria may precede changes in tight junctions in early stage of inflammation. Cytokines such as IL-22 and IL-18 produced by ENS and RORγt^+^ ILCs are decreased.

In addition to its own metabolic activities and interactions with immune responses, the metabolites produced by the gut microbiota also play an important role in maintaining the intestinal barrier. However, few large-scale clinical trials have been performed to confirm the role of these metabolites in the treatment of IBD. Further research on these metabolites may provide new options for IBD treatment (Fig. 1).

## Conclusion

IBD is often accompanied by dysbiosis, which can in turn aggravate intestinal inflammation. *F. prausnitzii*, *A. muciniphila*, *L. rhamnosus* L34, and other microorganisms regulate the intestinal barrier and reduce intestinal and systemic inflammation by decreasing intestinal permeability, increasing the expression of tight junction markers, and alleviating intestinal tissue injury and over-activated immune responses. Regarding the mechanical barrier, tight junction dysregulation is associated with chronic inflammation, and abnormal intestinal permeability precedes the onset of disease. Mucin is an important component of the intestinal mucus barrier. Mucin deficiency or genetic alterations affecting mucin function lead to increased susceptibility to IBD with dysbiosis. However, mucin expression gradually increases when patients are in remission. Therefore, changes in the intestinal barrier affect all stages of IBD.

The interactions among intestinal immunity (especially intestinal neuroimmunity), the gut microbiota, and the intestinal barrier are also indispensable to IBD. By reducing epithelial inflammatory responses, regulating the expression of cytokines such as IL-22, TNF-α, and IL-6, and restoring the composition of gut microbiota, exosomes participate in repairing and maintaining the integrity and function of intestinal barrier. Macrophages play an important role in intestinal neuroimmunity by maintaining the integrity of the intestinal barrier, regulating neuronal activity, and promoting an anti-inflammatory intestinal environment. As a tissue-protective cytokine that exclusively targets epithelium, the role of IL-22 in maintaining the intestinal epithelial barrier and regulating intestinal immunity is attracting increasing attention. On the one hand, IL-22 regulates the distribution of the gut microbiota, while on the other hand, the interaction between the gut microbiota and intestinal immune cells also affects the expression of IL-22 and reduces the susceptibility to inflammation. In addition, the gut microbiota interacts with its related metabolites and other components of the intestinal microenvironment to regulate the intestinal barrier and affect IBD.

Changes in the interplay between the intestinal barrier and the gut microbiota are particularly crucial for IBD. Further exploration of the interactions among the intestinal barrier, neuroimmune circuits, the gut microbiota, and its related metabolites will contribute to a deeper understanding of IBD and provide options for future treatment.

## Figures and Tables

**Table 1:** Changes in gut microbiota in IBD.

Phylum	Class	Order	Family	Genus	Species
Bacteroidete	Bacteroidetes UC↑^1^, CD↑^1^	Bacteroidales CD↓^4^	Rikenellaceae UC↑^1^		
			Bacteroidaceae	Bacteroides UC↑^1^	B.vulgatus CD↓^4^, B. caccae CD↓^4^
			Porphyromonadaceae	Odoribacter	
				CD↓^2^, IBD↓^2^	
				Parabacteroides CD↑^1^	
Firmicutes	Clostridia	Clostridiales CD↓^4^	Lachnospiraceae	Roseburia	R. intestinalis CD↓^4^
UC↓^1^, CD↓^5^				UC↓^2^, CD↓^3^, IBD↓^2^	
				Roseburia	R. hominis UC↓^6^
				UC↓^2^,CD↓^3^,IBD↓^2^	
				Lachnobacterium	
				UC↑^1^	
				Coprococcus	C. comes CD↓^4^
			Clostridiaceae CD↓^1^	Clostridium	C. nexile CD↓^4^, C. bolteae CD↓^4^
				UC↑^2^, CD↑^2^	
					C. leptum IBD↓^8^
				Blautia	B. hanseni CD↓^4^
					B. coccoides IBD↓^8^
				Butyricicoccus	B. pullicecorumIBD↓^10^
			Ruminococcaceae	Ruminococcus CD↓^1^	R. gnavus UC↑^3^, R. gnavus CD↓^4^,
			CD↓^1,3^ CD↑^2^		R.torques CD↓^4^
			Ruminococcaceae	Faecalibacterium	F. prausnitzii UC↓^3,6^ CD↓^4,5,8^
				CD↓^1,11^, CD↑^2^, UC↓^9^	
			Peptococcaceae	Phascolactobacterium	
			CD↓^1^	UC↓^2^, CD↓^3^	
			Eubacteriaceae	Eubacterium	E. rectale UC↓^3^, CD↓^4^
	Bacilli	Lactobacillales	Leuconostocaceae		
			UC↓^3^		
			Lactobacillaceae	Lactobacillus IBD↑^7^	
		Gemellales	Gemellaceae	Gemella	G. morbillorum CD↑^4^
	Negativicutes	Selenomonadales	Veillonellaceae	Veillonella	V. parvula CD↑^4^
Actinobacteria	Actinobacteridae	Bifidobacteriales	Bifidobacteriaceae	Bifidobacterium	B. bifidum CD↓^4^, B. longum CD↓^4^,
CD↓^1^				CD↓^1^, IBD↑^7^	B. adolescentis CD↓^4^, B. dentum CD↓^4^
Proteobacteria	Gammaproteobacteria	Enterobacteriales	Enterobacteriaceae	Escherichia CD↑^2, 11^	E. coli UC↑^3^, CD↑^4^, IBD↑^8^
UC↑^1^, CD↑^1^			CD↑^1,2,4^		
				Shigella CD↑^2, 11^	
		Pasteurellales	Pasteurellaceae	Hemophilus (spp.)	H. parainfluenzaeCD↑^4^
			CD↑^4^		
	Betaproteobacteria	Neisseriales	Neisseriaceae	Eikenella	E. corrodensCD↑^4^
	Deltaproteobacteria	Desulfovibrionales	Desulfovibrionaceae	Bilophila	B. wadsworthiaUC↓^3^
Fusobacteria	Fusobacteria	Fusobacteriales	Fusobacteriaceae	Fusobacterium	F. nucleatum UC↑^3^, CD↑^4^
			CD↑^4^		
Tenericutes	Mollicutes IBD↓^12^	Anaeroplasmatales IBD↓^12^	Anaeroplasmataceae	Asteroleplasma IBD↓^12^	
CD↓^1,12^			IBD↓^12^		
Tenericutes	Mollicutes IBD↓^12^	Anaeroplasmatales IBD↓^12^	Anaeroplasmataceae	Asteroleplasma IBD↓^12^	
CD↓^1,12^			IBD↓^12^		

*Annotations: superscript numbers 1–12 refer to the following studies: 1. Imhann, Vich Vila *et al*. 2018; 2. Morgan, Tickle *et al*. 2012; 3. Knoll, Forslund *et al*. 2017; 4. Gevers, Kugathasan *et al*. 2014; 5. Sokol, Pigneur *et al*. 2008; 6. Machiels, Joossens *et al*. 2014; 7. Wang, Chen *et al*. 2014; 8. Duboc, Rajca *et al*. 2013; 9. Varela, Manichanh *et al*. 2013; 10. Eeckhaut, Machiels *et al*. 2013; 11. Thorkildsen, Nwosu *et al*. 2013; 12. Willing, Dicksved *et al*. 2010.
